# Sex differences in postnatal weight gain trajectories of extremely preterm newborns

**DOI:** 10.1038/s41372-021-01099-2

**Published:** 2021-05-25

**Authors:** Fu-Sheng Chou, Hung-Wen Yeh

**Affiliations:** 1 Division of Neonatology, Department of Pediatrics, Loma Linda University, Loma Linda, CA USA; 2 Division of Health Services and Outcomes Research, Children’s Mercy-Kansas City, Kansas City, MO USA; 3 School of Medicine, University of Missouri-Kansas City, Kansas City, MO USA

**Keywords:** Risk factors, Paediatrics

## Abstract

**Objective:**

Both postnatal growth and sex play a crucial role in long-term outcomes of extremely preterm newborns (EPNs), but the relationship between sex and postnatal growth is not clear. This study aims to assess sex differences in weight trajectories.

**Study design:**

Weight data in the first 200 days of life from 4327 EPNs were used for generalized additive mixed modeling. We considered gestational age and sex as fixed-effects, and included random intercepts and random slopes for postnatal age. We assessed interactions between fixed-effects and postnatal age.

**Results:**

Male EPNs had higher predicted weight trajectories than females. Weight z-score trajectories decreased in both sexes before term-equivalent age comparably, but females showed faster increases afterward. Although weight gain velocity was comparable between both sexes, weight gain velocity in male EPNs was lower compared to the corresponding reference values from the 2013 Fenton growth charts, which explained slower z-score rises.

**Conclusion:**

Sex disparity exists in postnatal weight gain trajectories of EPNs after reaching the term-equivalent age.

## Introduction

Debate on what constitutes as the most optimal weight gain and overall growth among extremely preterm newborns (EPNs) in the early postnatal period continues to receive significant attention among the clinical research communities. The current goal backed by the American Academy of Pediatrics (AAP) Committee on Nutrition aims to achieve a growth rate that is similar to the growth rate of an uncomplicated growing fetus [1]. Although studies suggest that relatively uncomplicated preterm newborns were able to achieve a fetal growth rate in at least the first few weeks of life after the initial weight-loss period after birth, up to 50% of EPNs still failed to achieve expected weight at term-equivalent age (36–40 weeks postmenstrual age). This failure occurred despite rigorous postnatal nutrition programs and thus was labeled as postnatal growth failure (PGF) [2–7]. EPNs may simply follow a weight gain pattern distinct from their fetal counterparts due to extremely preterm birth [2, 3]. Factors that influence postnatal weight gain include free water contraction, the severity of illness, comorbidities of extreme prematurity, and the contemporary clinical approaches to parenteral and enteral nutrition. As oral skills and thermoregulation are highly energy-intercorrelated, the delay in reaching autonomic function maturity also affects weight gain. A recent report also suggested that antenatal factors such as placental insufficiency may influence postnatal weight gain trajectories in extremely low birth weight (ELBW) newborns [8, 9]. While it is crucial to understand the role of postnatal nutrition in the overall growth and developmental outcomes of EPNs, it is equally important to examine contributions from non-nutritional factors.

Sex disparities in fetal and child growth and the roles of sex differences in preterm neonatal outcomes have been well established in the literature [10, 11]. Studies also suggested the existence of sex-specific hormone responses to intrauterine and postnatal stress [11]. Male fetuses develop greater mass soon after conception and are more sensitive to hormonal regulations. In contrast, female fetuses are more prone to tight regulation of homeostasis in order to achieve survival benefit [11]. As opposed to the intrauterine growth of uncomplicated fetuses, EPNs represent a group of vulnerable “sick fetuses” forced to grow and develop in a hostile *ex utero* environment. Although *ex utero* growth and sex have both been individually linked to neonatal outcomes of EPNs, the association between growth and sex is not entirely clear. Attempts to understand EPN growth overall and whether sex plays a role in *ex utero* growth and development of EPNs using longitudinal weight data have been made and reported. A summary of recent reports on this topic is available in Supplemental Table [12–16]. These reports assumed linearity of weight gain with age, which does not reflect the true non-linear nature of weight changes during different stages of illness and development. The assumption of linear weight gain may miss the opportunity to capture a dynamic interacting relationship between sex and age.

The role of sex in human growth and development is best exemplified by our routine use of sex-specific growth charts. The widely used 2013 Fenton growth charts and the Intergrowth-21^st^ growth charts for EPNs, however, were developed to have female and male curves constructed separately. It remains a question whether sex-specific growth trajectory models for EPNs are superior to unisex models, and whether EPNs of opposing sex truly follow different growth trajectories [17, 18].

In this study, we hypothesize that male and female EPNs follow distinct postnatal weight gain trajectories that are different from each other and from the corresponding weight-at-birth standards (the 2013 Fenton growth charts). We took advantage of the generalized additive mixed modeling (GAMM) methodology without assuming linearity of weight trajectories while taking into account within-patient dependency. By taking this approach, we aimed to study the weight trajectories of EPNs and investigate the dynamic interaction between sex and postnatal age. To this end, we performed weight trajectory modeling using a retrospective dataset derived from de-identified electronic health records (EHR), followed by comparison to the 2013 Fenton sex-specific growth charts [17].

## Methods

### Study design, data source, and study population

We conducted a retrospective longitudinal observational study using data records from the Cerner Health Facts^®^ data warehouse which contained the Health Insurance Portability and Accountability Act (HIPAA)-compliant de-identified patient records since 2000. The Health Facts^®^ database was compiled by Cerner using electronic health records from participating academic and private facilities in the United States and available to contracted academic institutes for research purposes only. One data scientist at Children’s Mercy Children’s Research Institute accessed the database [19]. The database (accessed February 2019) contained over 500 million records from over 180 million patients across 664 facilities in the United States. Health Facts^®^ data was waived from requiring mandatory review for human subject research by Children’s Mercy Children’s Research Institute Office of Research Integrity.

**Fig. 1:**
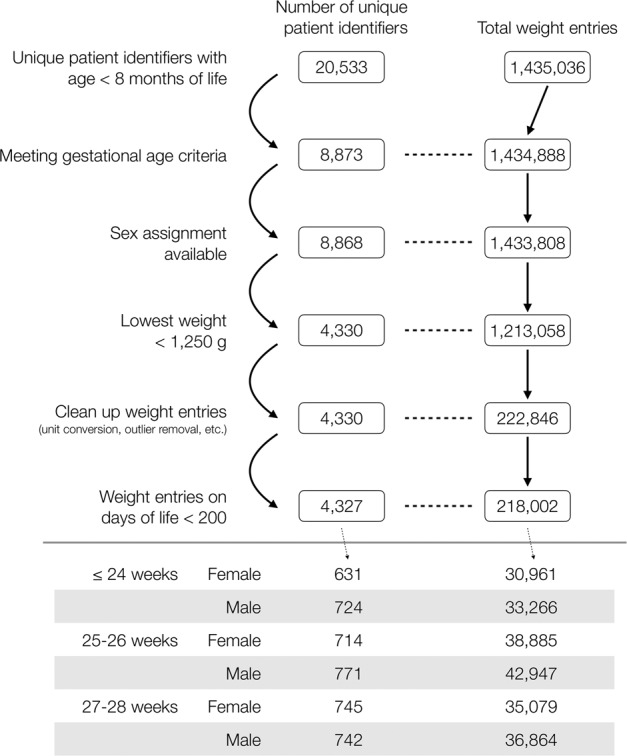
Data clean-up process and final numbers of patients and weight entries. A flow chart depicting the dataset clean-up process and the resultant number of unique patient identifiers as well as the number of corresponding weight entries.

Three steps were taken during the initial cohort selection process by the data scientist: 1) extract encounters created at less than eight months of age with a diagnosis corresponding to a birth gestational age (GA) of 28 weeks or less; 2) identify unique patient identification numbers (PINs) associated with the encounters extracted in the previous step, followed by extracting all encounters associated with the identified PINs; 3) construct a diagnosis table and a weight entry table from all encounters extracted in the previous step. This process resulted in 8873 unique patient identifiers and over 1.4 million weight entries (Fig. 1). Records were further excluded if opposing sex assignment was found in different encounters or if there was missing or no sex assignment. Only patients with a minimum body weight of less than 1250 g between day of life (DOL) 0 and 7 were included. All weight entries that were negative, zero, or larger than 10,000 g were excluded. A visual depiction of the data that went through the clean-up process, with resultant numbers of unique patient identifiers and weight entries, is available in Fig. 1.

### Estimation of date of birth, and day of life, and postmenstrual age

The Health Fact^®^ dataset variables that were available to us did not include the date of birth (DOB) due to compliance requirements, and all the dates in the dataset were shifted to protect privacy. Because the age (in days) of the infants when each weight record was entered was crucial to our analysis, a critical part in our study was to establish an anchor date for each unique patient identifier to validate weight entries. To this end, we back-calculated to estimate DOB (denoted as “est-DOB”, used to serve as an anchor date) for each unique patient identifier by subtracting the age (in days) of each patient from the encounter start date, with the knowledge that the “est-DOB” would not be the real DOB. Discrepancies in “est-DOB” among different encounters were resolved by using “est-DOB” calculated from the encounter created at the patient’s youngest age. After “est-DOB” was assigned to each patient, the DOL for each weight entry was calculated by subtracting “est-DOB” from the date of weight data entry. Those entries with a negative DOL were excluded.

### Gestational age (GA) assignment

GA may play a role in postnatal weight trajectory, as the likelihood of neonatal comorbidities of extreme prematurity is associated with GA. GA is not part of the dataset but was available as International Classification of Diseases, 9th and 10th revisions, Clinical Modifications (ICD-9-CM, ICD-10-CM) codes: GA 24 weeks and less (ICD-9-CM: 765.21, 765.22; ICD-10-CM: P07.21, P07.22, P07.23), GA 25 and 26 weeks (ICD-9-CM: 765.23; ICD-10-CM: P07.24, P07.25), and GA 27 and 28 weeks (ICD-9-CM: 765.24; ICD-10-CM: P07.26, P07.31). Data clean-up processes were performed to ensure that, if conflicting diagnosis codes for birth GA existed, the patient was assigned to the GA group based on the priority of the diagnosis codes.

### Data analysis

Data clean-up, statistical analyses, and modeling of weight gain trajectory were performed in R 3.6.3 and RStudio 1.2 [20, 21]. All codes are available upon request. After data clean-up, 4327 unique EPNs across 86 facilities, with a total of 218,002 weight entries, were identified. Descriptive statistics were performed to characterize inter-group variations of selected variables. As there were varying sample sizes across sex and GA groups with a dependence of p-values on sample sizes, Cohen’s W was used to assess the effect size of categorical variables.

Weight entries between DOL 0 and 199 were used for modeling. Patients may not all have daily weight available for use in modeling. Nonetheless, the majority of patients had more than 10 weight entries, with some having more than 100 weight entries to contribute to the model (Supplementary Fig. 1A). Additionally, there were more than 10 weight entries for most days, and there were more than 100 weight entries for each day in the first 100 days of life in all groups (Supplementary Fig. 1B).

The GAMM was applied to model each of weight and weight z-score trajectories. GAMM includes both fixed- and random-effects as linear mixed-effects models (LMM) do [8], with the exception that fixed-effects include, in addition to parametric effects as in LMM, a non-linear non-parametric smooth function of postnatal age (DOL) which relaxes the linearity assumption. For this work, we used random intercepts and random slopes for postnatal age in DOL as random-effects in GAMM to account for within-patient dependence. We considered 6 GAMM with the same aforementioned random-effects, but various combinations of fixed-effects: 3 (sex, GA group, and sex-by-GA) in the parametric part only, and 3 in both parametric and non-parametric parts. A GAMM with only parametric sex effect would suggest the same trajectory pattern for both sexes while the trajectory of one sex would be higher than the other by the same difference across the 200-day period (i.e., parallel trajectories); on the other hand, a GAMM with parametric and non-parametric sex effects would suggest that each sex has their own trajectory (or equivalently, a sex-by-smooth function of DOL interaction, i.e., sex modifies growth trajectories). GAMM were built using the *gamm4* package [22]. For weight z-score modeling, DOL 0 was transformed into GA 24 weeks 0 day, 26 weeks 0 day, 28 weeks 0 day for the groups born at GA 24 weeks and less, GA 25 and 26 weeks, and GA 27 and 28 weeks, respectively, and z-scores were calculated using the 2013 Fenton growth charts [17]. The optimal model among the 6 GAMM was then selected by the Akaike information criterion (AIC). As a post hoc analysis, likelihood ratio test (LRT) was applied to compare GAMM with vs. GAMM without non-parametric sex effects (i.e., whether sex modified growth trajectory) within each GA group. Model parameters were estimated using restricted maximum likelihood estimation.

## Results

Among 4327 unique EPNs across 86 facilities, there were 1355 (31.3%), 1485 (34.3%), and 1487 (34.4%) EPNs in the groups born at GA 24 weeks and less, GA 25 and 26 weeks, and GA 27 and 28 weeks, respectively, 51.7% (*n* = 2,237) of whom were male (Table 1). The distributions of race/ethnicity and comorbidities appeared similar between the two sex groups for each GA subgroup, as suggested by between-sex Cohen’s W all being <0.1, a small effect according to Cohen’s guideline [23].

**Table 1:** Demographic information.

Comorbidity	GA 24 weeks and less			GA 25 and 26 weeks			GA 27 and 28 weeks		
Sex	Female	Male	Effect size^a^	Female	Male	Effect size^a^	Female	Male	Effect size^a^
Number	631 (46.6)	724 (53.4)	-	714 (48.1)	771 (51.9)	-	745 (50.1)	742 (49.9)	-
Race/ethnicity									
White	239 (37.9)	279 (38.5)	0.05	282 (39.5)	321 (41.6)	0.03	369 (49.5)	343 (46.2)	0.06
Black	226 (35.8)	262 (36.2)		247 (34.6)	259 (33.6)		208 (27.9)	223 (30.1)	
Hispanic	19 (3.0)	29 (4.0)		17 (2.4%)	22 (2.9%)		22 (3.0%)	15 (2.0%)	
Asian/Pacific Islander/Native American	10 (1.6)	16 (2.2)		17 (2.4)	15 (1.9)		24 (3.2)	20 (2.7)	
Other/Unknown	137 (21.7)	138 (19.1)		151 (21.1)	154 (20.0)		122 (16.4)	141 (19.0)	
Comorbidity									
IVH - low grade	89 (14.1)	112 (15.5)	0.02	106 (14.8)	119 (15.4)	0.01	87 (11.7)	90 (12.1)	0.01
IVH - high grade	106 (16.8)	156 (21.5)	0.06	77 (10.8)	109 (14.1)	0.05	40 (5.4)	46 (6.2)	0.02
NEC^b^	28 (4.4)	59 (8.1)	0.08	39 (5.5)	55 (7.1)	0.03	21 (2.8)	34 (4.6)	0.05
BPD	285 (45.2)	322 (44.5)	0.01	344 (48.2)	404 (52.4)	0.04	258 (34.6)	284 (38.3)	0.04
ROP - low grade	118 (18.7)	152 (21)	0.03	195 (27.3)	191 (24.8)	0.03	155 (20.8)	162 (21.8)	0.01
ROP - high grade	85 (13.5)	76 (10.5)	0.05	73 (10.2)	61 (7.9)	0.04	19 (2.6)	23 (3.1)	0.02
PVL	38 (6.0)	52 (7.2)	0.02	33 (4.6)	35 (4.5)	0.00	25 (3.4)	20 (2.7)	0.02

Data presented as number (percentage).

*GA* gestational age, *IVH* intraventricular hemorrhage, *NEC* necrotizing enterocolitis, *BPD* bronchopulmonary dysplasia, *ROP* retinopathy of prematurity, *PVL* periventricular leukomalacia.

^a^Cohen’s W test.

^b^Stage 2 or 3.

**Fig. 2:**
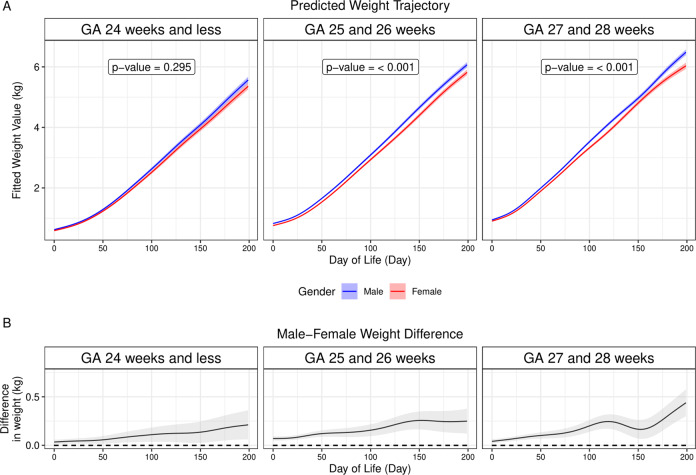
Generalized additive mixed modeling of postnatal weight trajectories in extremely preterm newborns. **A** Predicted weight trajectories for male (blue) and female (red) extremely preterm newborns were plotted against the postnatal age (in days of life), with shades representing 95% confidence interval (CI). Likelihood ratio tests were used for significance testing (refer to text for details), with results showing that, while there was no significant difference in the group born at GA 24 weeks or less, the differences were significant in the groups born at GA 25 and 26 weeks as well as at GA 27 and 28 weeks. **B** Weight trajectory difference with 95% CI (shaded areas) between male and female (male minus female) was plotted against the postnatal age (in days of life). Note the dashed line represents zero differences in weight trajectories. The figure showed that predicted weight trajectories in male infants were higher than those in female infants. GA stands for gestational age.

**Fig. 3:**
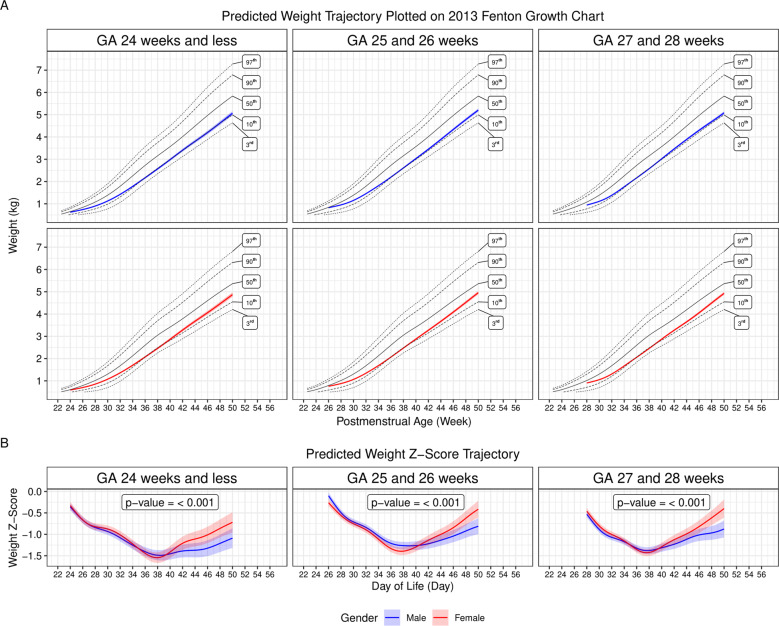
Generalized additive mixed modeling of postnatal weight z-score trajectories in extremely preterm newborns. **A** Predicted weight trajectories for male and female extremely preterm newborns (EPNs) were plotted on the 2013 Fenton sex-specific growth charts to compare longitudinal weight gain trajectories of the EPNs to the referenced weight-at-birth growth charts which represent intrauterine growth. **B** Predicted weight z-score trajectories in male and female EPNs were plotted against postmenstrual age (PMA). Likelihood ratio tests showed significant differences in weight z-score trajectories between male and female in all three GA groups. For both **A** and **B**, the blue (male) and red (female) lines represent predicted values of the weight (**A**) and weight z-score (**B**) trajectories. The shaded areas represent the 95% confidence intervals.

AIC suggested a GAMM including both GA and sex as the parametric fixed-effects as well as their interactions with smooth function of DOL for the weight trajectory, suggesting that both GA and sex modified postnatal weight trajectories. Predicted weight trajectories in male EPNs were higher than those in female EPNs for all three GA groups (Figs. 2A and 3). The differences increased with DOL (Fig. 2B), similar to the differences observed in male and female fetuses. Of note, LRT suggested the role of sex in weight trajectories was significant in the groups born at GA 25–26 weeks and at GA 27–28 weeks but not in the group born at GA 24 weeks and less (Fig. 2A).

We next mapped the predicted weight trajectories from the GAMM to the 2013 Fenton sex-specific growth charts. Visual inspection revealed between-sex discrepancy in weight z-score trajectories after reaching the term-equivalent age. We then modeled weight z-score trajectories up to 50 weeks postmenstrual age  (PMA) for each GA group using the same modeling approach as for the weight outcome. We found that both male and female EPNs had a decline in weight z-scores from birth to term-equivalent age at similar rates (Fig. 3B), resulting in nadirs at around term-equivalent age (Table 2). After the nadirs, weight z-scores of female EPNs increased steadily and rapidly in all three GA groups. On the other hand, z-score increases following the nadirs in male EPNs were relatively slow and inconsistent. Significance tests showed that the weight z-score trajectories were significantly different between male and female infants in each of the three GA groups. These findings suggested the existence of sex disparities in weight accrual between EPNs and the referenced values after reaching the term-equivalent age, resulting in faster z-score rises in female EPNs.

**Table 2:** Lowest predicted z-scores and the corresponding postmenstrual age (PMA) from the predicted weight z-score trajectories in each of the sex and gestational age (GA) groups.

	Female		Male	
GA group	PMA	Lowest z-score	PMA	Lowest z-score
GA 24 weeks and less	37 weeks 6 days	−1.55	38 weeks 3 days	−1.49
GA 25 and 26 weeks	37 weeks 5 days	−1.40	38 weeks 6 days	−1.27
GA 27 and 28 weeks	37 weeks 4 days	−1.43	37 weeks 5 days	−1.38

**Fig. 4:**
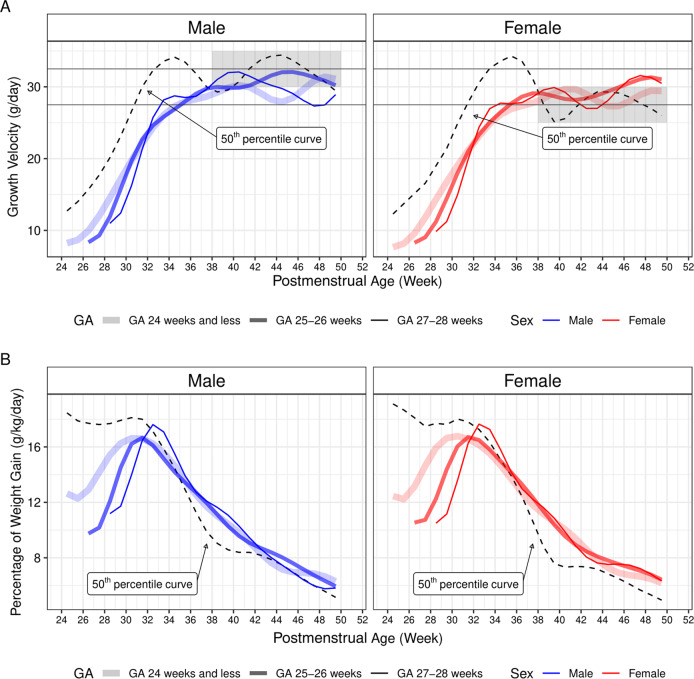
Comparing predicted weight gain velocity and the percentage of weight gain to the growth standards. **A** Weight gain velocities were plotted against gestational age (GA). The gray rectangles in the plotting areas encompass the upper and lower limits of the reference growth velocities (dashed lines) for sex within the indicated postmenstrual age (PMA) range (38–50 weeks). Solid blue (male) and red (female) lines indicate predicted growth velocities in the extremely preterm newborns. **B** Percentages of weight gain for male (blue) and female (red) were plotted against PMA. Note that, for both **A** and **B**, the reference weight gain velocity lines (dashed lines) were derived from the 2013 Fenton growth chart 50^th^ percentile lines for the corresponding sexes. Additionally, weight gain velocity in grams per day (**A**) was generated by calculating the weight difference between two consecutive days, followed by taking a 7-day average; percentage of weight gain (**B**) was calculated by dividing weight gain velocity (in grams per day) by the weight of the first of the two consecutive days, followed by taking a 7-day average. The combinations of the thickness and the degree of transparency of the solid lines represent corresponding GA groups, with the thinnest and least transparent lines representing the group born at GA 27–28 weeks, and the thickest and most transparent lines representing the group born at GA24 weeks and less. These solid lines are color coded to present female (red) and male (blue).

We then calculated weight gain velocity (in the unit of g/day) for each sex in all three GA groups using the predicted values from the best GAMM of weight trajectories. In the model, we did not observe any initial weight loss. Weight gain velocity increased as postnatal age increased, reaching ~30 g/day at the term-equivalent age, in all three GA groups irrespective of sex (Fig. 4A). When compared to the reference curves derived from the 50^th^ percentile reference growth curves of the 2013 Fenton growth charts, weight gain velocity in EPNs were lower than the peak reference growth velocity (~34 g/day) before reaching the term-equivalent age in both sex groups. The fall in weight gain velocity between 36 and 40 weeks in the reference curves was not observed in EPNs. After the term-equivalent age, the predicted weight gain velocity was similar between male and female EPNs, of ~27.5–32.5 g/day. This velocity was visually lower than the 50^th^ percentile reference curve (of ~30–35 g/day) in male but slightly higher than the 50^th^ percentile reference curve (of ~25–30 g/day) in females (Fig. 4).

When the percentage of daily weight gain (in the unit of g/kg/day) was plotted against PMA, an initial acceleration phase was observed for each GA group in both sexes before GA 31–34 weeks, reaching a peak of 16–18 g/kg/day. This period was preceded by a decrease (in the group born at GA 24 weeks and less) or a slow increase (in the groups born at GA 25 and 26 weeks and at GA 27 and 28 weeks) in the first two weeks of life. The acceleration phase was followed by a deceleration phase, with no obvious sex differences noted. Nonetheless, sex differences were observed when comparing the predicted percentage of weight gain in EPNs to the percentage of weight gain in the reference curves after reaching the term-equivalent age. Specifically, female EPNs, but not males, showed persistently higher percentages of weight gain when compared to their references. The discrepancy was due to a prolonged decline in the percentage of weight gain in the reference curve between GA 38–40 weeks in females, which translates into a greater decrease in growth velocity in the same gestation period (compare dashed lines in Fig. 4A).

## Discussion

To our knowledge, this is the first report that described weight gain trajectories of EPNs in the early postnatal period using a complex modeling algorithm that did not require linearity assumption of the time-dependent weight measurements. By taking this approach, we described a model that recapitulates the dynamic nature of weight trajectories and growth velocity as a smooth function of DOL. This approach was different from the techniques used in earlier studies which took snapshots of weight measurements at predetermined time points, assuming linearity of weight gain trajectories, and calculating average weight gain velocity without taking into consideration different stages of physiological development (Supplemental Table) [12–16].

Using the same modeling technique, we further modeled the trajectories of normalized weight measurements using the 2013 Fenton growth charts as the normalization guides. Our findings were consistent with other published reports, suggesting a distinct weight gain trajectory pattern among EPNs that is different from intrauterine growth [2, 3, 6, 8]. The 2013 Fenton growth charts, although widely used, were constructed based on weight-at-birth to recapitulate intrauterine growth. Ideally, EPNs should follow the intrauterine growth trajectories with postnatal intensive nutritional programs, fulfilling the goal set forth by the AAP. Realistically, fluid contraction, acuity of illness, comorbidities, and nutritional routes all played a part in the trajectory of postnatal weight gain/loss, which is a reflection of the net summary of anabolism and catabolism and is dependent on multiple physiological and/or pathophysiological activities that are taking place at the same time. The result is a gradual decrease in weight z-score starting from birth, which gradually stabilizes when enteral nutrition was fully established. Subsequently, with the initiation of oral feeding around PMA 34–36 weeks, weight z-scores decrease again [9]. The dynamic changes in weight z-scores were faithfully captured in the models presented here. These changes showed a two-step decrease in weight z-scores in both sexes in all three GA groups. Further, they reinforced the notion in the recent review article by Fenton et al. published in the *Journal* that extrauterine growth failure or PGF based on weight z-score differences between birth and PMA 36–40 weeks may not convey long-term clinical significance [4, 5, 24]. In addition to comparing to intrauterine growth, EPNs may also benefit from their own sex-specific growth charts based on models that include the severity of illness, nutrition provision, presence or absence of comorbidities, among others, at least in the immediate postnatal period.

Although the nutrition protocols were not included in the dataset, we should safely assume that the studied patients of both sexes received comparable nutrition if they were cared for in the same facility, as current nutrition recommendations do not emphasize on a sex-stratified nutrition program. Therefore, it is reasonable that the predicted weight trajectories and weight gain velocities followed similar trends between the two sex groups from birth to term-equivalent age. Consistently, growth velocity in the reference growth charts was also similar between males and females before term-equivalent age. On the other hand, given a positive association between postnatal growth and improved neurodevelopmental outcomes, can male EPNs benefit from higher nutrition standards and goals to achieve comparable neurodevelopmental outcomes as their female EPN peers later in life? Will they tolerate higher nutrition goals?

Sex differences in weight z-score trajectories became apparent after the term-equivalent age, at a time when most EPNs have already been discharged home on full oral nutrition. Weight gain after term-equivalent age is most likely a result of on-demand feeding that is primarily dependent on appetite and gastric emptying speed. Comparing growth velocity between male and female reference curves, a greater decrease in growth velocity was seen in female between PMA 36 and 40 weeks in the reference curve; afterward, the velocity stayed lower in females (25–30 g/day, shaded area in Fig. 4A) than in males (30–35 g/day). However, in EPNs, we did not notice a qualitative difference between the two sexes (models derived from the GAMM technique are not amenable to a derivative calculation for statistical comparison). We found that the predicted maximum growth velocity in male EPNs was lower than the maximum growth velocity in the corresponding male reference curve, implying that male EPNs may not have received sufficient nutrition intake. Male EPNs may potentially benefit from a more aggressive nutrition program for a longer period, even after discharge.

When it comes to examining the association between post-discharge growth and neurodevelopmental outcomes, the literature showed conflicting results, although more studies suggested a positive relationship between post-term weight gain and neurodevelopmental outcomes in preterm infants [25–28]. Specifically, Belfort et al. reported that greater weight gain was associated with better neurodevelopmental outcomes in preterm infants born at less than 33 weeks in a multi-center Australian cohort of 613 infants [28]. From these observations, one may speculate whether faster regain in weight z-scores in female EPNs after term-equivalent age may account for better cognitive and motor development in female EPNs at later stages of life [10, 29, 30]. On the other hand, early life events have been shown to affect long-term health outcomes such as cardiometabolic disorders [31–33]. It is currently unknown whether or not an increased rate of regaining weight z-score after oral intake is fully established in female EPNs puts them at increased risk of adverse long-term outcomes.

In the 2013 revision of the Fenton growth charts, a cubic spline smoothing technique was introduced to improve the connection between fetal growth reference from GA 22 to 40 weeks and the sex-specific growth standards published by the World Health Organization between GA 40–50 weeks [17]. Inevitably, smoothing led to a reduction in the convexity of the reference curves and a potential to overestimate growth, although one may also argue that the smoothing procedure alleviated fetal growth reduction between GA 36–40 weeks that is experienced only by term newborns before birth [3]. Nonetheless, as implicated by the authors, size measurement and assignment based on the growth charts would only be valid between GA 22 and 36 weeks and at 50 weeks where curves are consistent with data [17]. Therefore, caution must be exercised when interpreting z-score trending directions and slopes around the term-equivalent age. In other words, although we were able to calculate z-score nadirs from the predicted curves based on the growth references (Table 2), the precise nadirs of z-scores for each sex and GA group may never be known.

The biggest difference between the original and the revised Fenton growth charts is in male growth curves after 36 weeks postmenstrual age, where a larger weight gain was seen in the revised version [17]. Using the original version of the growth chart, male EPNs would have had regained weight z-score after the term-equivalent age similar to their female counterparts. Whether the upward correction of the male growth curves to match WHO growth standards at 50 weeks of gestation has an impact on the nutrition goals and alters long-term outcomes of the male EPNs is not entirely clear. Understanding how growth charts are developed and optimally utilized in clinical settings is of utmost importance.

## Limitations

This study was subjected to limitations that are common to retrospective observational studies based on hospital EHR. First, we identified patients for inclusion based on ICD-9-CM and ICD-10-CM codes. Coding errors or conflicting codes in different encounters of a single patient may create potential errors in GA group assignment. Additionally, in the modeling of weight z-scores, we made assumptions to the mean GA in order to convert weight data into z-scores, which may not be accurate. Furthermore, weight measurement routines such as the timing of the day, subtraction of life support apparatus, and nutrition protocols may be variable at different facilities. Although random intercepts and random slopes were introduced at the individual level, institution-specific effects were not considered in our model. Moreover, maternal, fetal, and perinatal variables were not available for examination in terms of their contributions to postnatal weight trajectories. Finally, this study’s weakness is its lack of data on length and head circumference for modeling.

## Conclusion

We reported the role of sex in postnatal growth trajectories of the EPNs using a complex modeling approach. The GAMM methodology provides flexibility in studying weight gain trajectory in a non-linear fashion. Future studies may include additional demographic, maternal, fetal, and postnatal comorbidity variables, long-term neurodevelopmental and cardiometabolic outcome variables in the model for comparison, with an ultimate goal to identify the most optimal growth trajectory in the early postnatal period conferring a long-term health benefit in this vulnerable population.

## Supplementary information

Summary of approaches to postnatal weight trajectory modeling in the literature.

Summary of weight entry number based on patient group and day of life.
