# Supplementary material for: Sex differences in postnatal weight gain trajectories of extremely preterm newborns
Source: J Perinatol. 2021 May 25;41(8):1835–44. doi: 10.1038/s41372-021-01099-2 (PMC8342307; doi:10.1038/s41372-021-01099-2)
Supplement: Supplementary file 1 — Summary of approaches to postnatal weight trajectory modeling in the literature. [file 41372_2021_1099_MOESM1_ESM.pdf]

Supplemental Table. Summary of approaches to postnatal weight trajectory modeling in the literature

Publication	Year	Country	Number of Study Site	Number of Infants	Inclusion Criteria	Growth Standard Curve Used	Modeling Approach	Modeling type	Sex as a variable
Guo et al.[12]	1997	USA	Multiple	867	• VLBW ≤ 1,500 g<br>• LBW 1,501-2,500 g<br>• Exclude multiple gestation and several congenital anomalies (see reference for detail)	None used	• 6 measurement points: the original due date, 4, 8, 12, 18, 24, 30, and 36 months after the original due date.<br>• Descriptive statistics comparing sex difference at birth and the original due date.<br>• Individual linear regression modeling of longitudinal weight trajectories for each weight (LBW and VLBW) and sex (boys and girls) category.	Linear	Yes
Ehrenkranz et al.[13]	1999	USA	Multiple	1,660	• Birth weight 501-1,500 g<br>• Admitted to the NICHD NRN centers within 24 hrs of life<br>• Survive > 1 week	Fetal growth reference (Alexander et al., 1996) - for comparing postnatal weight trajectories of preterm infants in each birth gestation category to the fetal growth at corresponding gestational ages.	• Weight measurement until reaching 2,000 g, transferred, discharge, or reaching 120 days of life.<br>• Daily weight measurement until regaining birth or 14 days of life; weekly measurement thereafter.<br>• Grouped by 100-g birth weight intervals (501-600 g, 601-700 g, etc.), or by 200-g intervals (501-700, 701-900, etc.) stratified by major comorbidities, or by gestational age category (24-25, 26-27, and 28-29 weeks GA), for mixed-effects modeling.<br>• Modeling with both sexes combined.	Linear	No
Cole et al.[14]	2014	UK	Multiple	4,973 (longitudinal growth curve)	• < 32 weeks GA	British 1990 birth weight reference	• Superimposition by Translation and Rotation random-effects modeling with both sexes combined.<br>• Derive weight gain in absolute (g/day) and relative (g/kg/day) terms.	Linear	No
Villar et al.[15]	2015	Multiple	Multiple	201	• 26-36 weeks GA without congenital anomalies, fetal growth restriction, and severe postnatal morbidity	None used	• Multi-step approach leading to a multilevel second-degree fractional polynomial mixed-effects model.<br>• Sex was included as a fixed-effect variable in the model.	Linear	Yes
Zozaya et al.[16]	2019	Spain	Multiple	21,825	• < 1,500 g<br>• 24 to 31 6/7 weeks GA<br>• No major congenital malformations	• 2013 Fenton growth charts for PMA < 50 weeks<br>• WHO growth charts for PMA ≥ 50 weeks	• Measurements were obtained at birth and at discharge/death<br>• Linear regression modeling of measurement changes or measurement z-score changes between discharge/death and birth; various comorbidities and sex as covariates.<br>• Average growth velocity was calculated by $\frac{Discharge/\,Death\,weight - Birth\,weight}{Length\,of\,stay}$	Linear	Yes
