# Supplementary material for: Sex differences in postnatal weight gain trajectories of extremely preterm newborns
Source: J Perinatol. 2021 May 25;41(8):1835–44. doi: 10.1038/s41372-021-01099-2 (PMC8342307; doi:10.1038/s41372-021-01099-2)
Supplement: Supplementary file 2 — Summary of weight entry number based on patient group and day of life. [file 41372_2021_1099_MOESM2_ESM.pdf]

Supplemental Figure 1

A

Number of Patients for Each Weight Entry Number Group

Weight Entry Number group	GA 24 weeks and less		GA 25 and 26 weeks		GA 27 and 28 weeks	
	Female	Male	Female	Male	Female	Male
≤ 10	234	312	147	179	144	155
11-100	275	273	459	482	562	537
> 100	122	139	108	110	39	50
Total	631	724	714	771	745	742

B

Number of Weight Entry for Each Day of Life

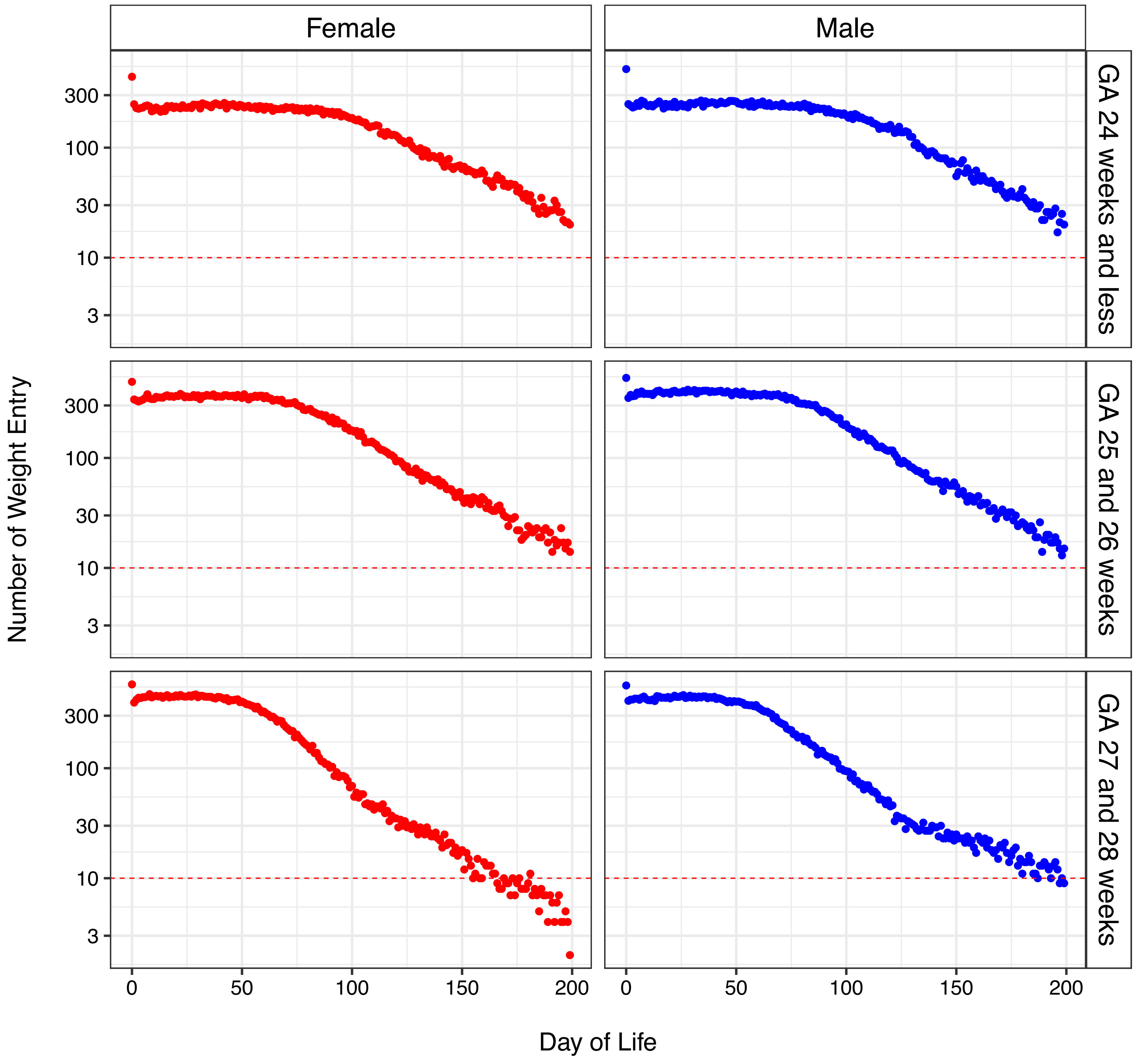

Supplemental Figure 1. (A) Number of patients (unique patient identifiers) for each weight entry number group. (B) Number of weight entries for each day of life. GA: Gestational age.
